# Photothermal Perylene Bisimide Hydrogels

**DOI:** 10.1002/chem.202300663

**Published:** 2023-05-10

**Authors:** Lisa Thomson, Rebecca E. Ginesi, Daniel D. Osborne, Emily R. Draper, Dave J. Adams

**Affiliations:** ^1^ School of Chemistry University of Glagsow G12 8QQ Glasgow UK

**Keywords:** gel, multicomponent, PBI, photothermal, radical anion

## Abstract

Gels formed using a perylene bisimide (PBI) as a low molecular weight gelator can show the photothermal effect. Formation of the PBI radical anion results in new absorption bands forming, meaning that subsequent irradiation with a wavelength of light overlapping with the new absorption band leads to heating of the gel. This approach can be used to heat the gel, as well as the surrounding milieu. We show how we can use electrochemical methods as well as multicomponent systems to form the radical anion without the need for UV light, and how we can use the photothermal effect to induce phase transitions in the solutions above the gels by exploiting photothermal behavior.

## Graphical abstract

In this paper, perylene bisimide gels are prepared that can exhibit the photothermal effect. This can be used for example to heat a solution above the gel.

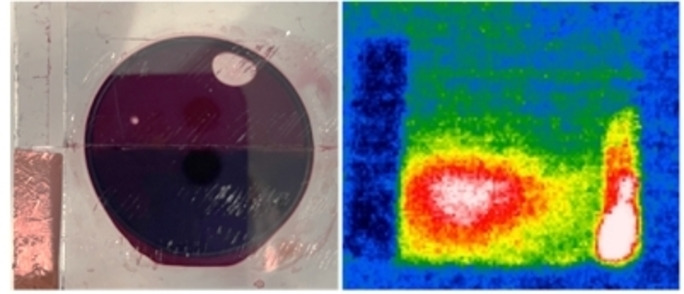

## Introduction

The photothermal effect is where heat is generated by irradiation with light.^[1]^ Other processes can occur such as fluorescence, but for effective photothermal behavior, these are minimized, and all the light energy is converted to heat. An exciting opportunity is to prepare materials where the photothermal effect will only take place at specific times or locations. To do this, a material needs to be designed so that it does not absorb light of a specific wavelength, and so there is usually no heating. If the material can be changed controllably such that light is then absorbed and the photothermal effect occurs, this should allow us to control when and where the heating occurs. Heating could be across the whole of the material, or in spatially specific positions.

A range of different materials have been used for photothermal heating. These include a range of inorganic materials^[2]^ including gold nanorods^[3]^ as well as a number of organic compounds.^[4]^ There has been recent interest in supramolecular materials which show the photothermal effect.^[5]^ A number of perylene bisimides (PBIs) have been used,^[6]^ especially in host–guest complexes which maintain the monomeric, non‐aggregated state of the PBIs.^[7]^ Reducing aggregation of the PBIs has been stated as a requirement for effective materials to be formed due to a lack of stability of the PBI radical anion.[^5^, ^8^] However, there are successful examples of solutions of peptide‐functionalized PBIs which aggregate in water which can successfully exhibit photothermal behavior.^[9]^

PBIs form a radical anion when reduced, followed by a second reduction to the dianion. Formation of the radical anion changes the absorption spectrum significantly, critically with new bands appearing in the near infra‐red (NIR) in some cases. The exact wavelengths of the new bands depend on the molecular structure and aggregated state. Hence, when in the ground state, these molecules do not absorb in the NIR, but when reduced to the radical anion, they do absorb and the photothermal effect can take place. Reduction to the radical anion can be carried out using light,^[10]^ chemically,^[11]^ electrochemically,^[12]^ or using E. Coli.^[7a]^

**Figure 1:**
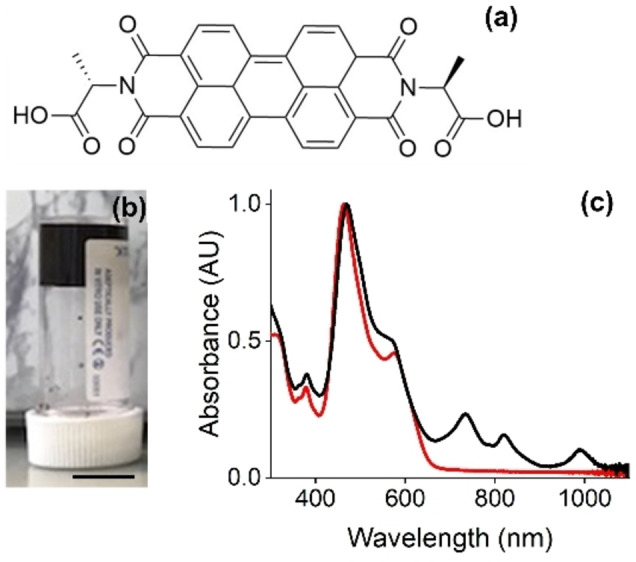
(a) Chemical structure of PBI‐A; (b) Photograph of a gel formed from PBI‐A in 7 mL Sterilin vial. Scale bars represent 1 cm; (c) Normalized UV‐Vis spectrum of a gel formed from PBI‐A before (red) and after (black) irradiation with a 365 nm LED for 10 min.

We have prepared hydrogels from PBIs, for example PBI‐A (Figure 1a) where the imide positions are functionalized with L‐alanine.[^10^, ^13^] These are inexpensive, easy to synthesize molecules that self‐assemble in water to give self‐assembled 1D aggregates by ordered stacking. Gels are formed when these one‐dimensional structures entangle and cross‐link, immobilizing the solvent and forming a gel (Figure 1b).^[10]^ The type of aggregate and the gel properties can be controlled by molecular structure and the gelation conditions. We have previously found that the radical anion formed from PBI‐A in the gel state lasts for many hours, and (due to the self‐assembled structure) is air‐stable.^[10]^

## Results and Discussion

Gels were prepared by dissolving PBI‐A at pH 9 at a concentration of 5 mg/mL. This solution was then acidified by the addition of glucono‐δ‐lactone (GdL) which slowly hydrolyses to gluconic acid.^[14]^ This slow pH change leads to homogeneous gels being formed (Figure 1b).[^10^, ^14b^] Irradiation with a 365 nm LED results in the formation of the radical anion, which can be seen by eye as a color change from dark red to purple, and results in new peaks in the UV‐vis absorption spectrum forming at 734, 821, and 991 nm (Figure 1c). The broad peak at 991 nm has been assigned previously to so‐called π‐dimers^[15]^ and whilst present for PBI‐A is not ubiquitous for such systems. For example, irradiation of the glycine‐derivatized PBI, PBI‐G, also results in the peaks indicative of the radical anion, with no significant new absorption peak formed in the region of 700 nm or greater (Figure S1, Supporting Information). As such, it is necessary to tune molecular structure and aggregates to enable gel formation where formation of the radical anion leads to peaks in the near‐IR.

**Figure 2:**
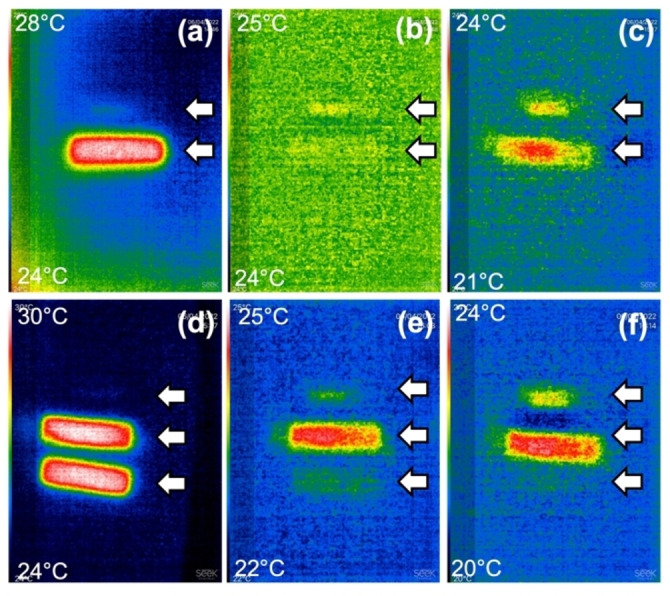
Photographs of gels formed from PBI‐A using a thermal camera. In all cases, the upper temperature achieved is at the top left (red) and the minimum temperature on each image in the bottom left (dark blue). (a) shows two gels (highlighted by arrows), one which has been irradiated with a 365 nm LED for 30 min (bottom) and one which has not (top). This cannot be seen however due to the background and cuvette being the same temperature; (b) shows the gels 2 min after stopping irradiation with the 365 nm LED showing the samples have now cooled to room temperature; (c) shows the cooled gels 10 min after irradiation with a 810 nm LED which has increased in temperature; (d) shows three gels (indicated by arrows), two which have been irradiated with a 365 nm LED for 30 min (middle and bottom) and one which has not (top). This cannot be seen due to the background and cuvette being the same temperature; (e) 2 min after the subsequent 810 nm irradiation on the middle gel but stopping all irradiation on the bottom gel; (f) 10 min after the subsequent 810 nm irradiation on the middle gel but stopping all irradiation on the bottom gel. Scale bars represent 1 cm.

For PBI‐A, in addition to formation of the radical anion, irradiation with a 365 nm LED also results in an increase in the temperature of the gel (Figure 2a and 2d). The temperature slowly decreases after the irradiation has stopped (Figure 2b), with the radical anion persisting for many hours after this. This temperature increase is simply due to the 365 nm LED, not the presence of the radical anion and, for example, occurs for the PBI‐G gel too (Figure S2). However, irradiating the gel containing the PBI‐A radical anion with an 810 nm LED (a wavelength which overlaps with the new absorption band created during the 365 nm irradiation) once the gel has cooled post‐irradiation with the 365 nm LED results in the gel re‐warming (Figure 2c). The PBI‐A gel alone is thermally stable until at least 70 °C (Figure S3). The second heating is due to the presence of the radical anion and does not occur for the corresponding PBI‐G sample.

Immediate irradiation with the 810 nm LED after the 365 nm irradiation results in the temperature of the gel being maintained as opposed to cooling to ambient occurring (Figure 2d–f), showing that the photothermal effect is in operation.

**Figure 3:**
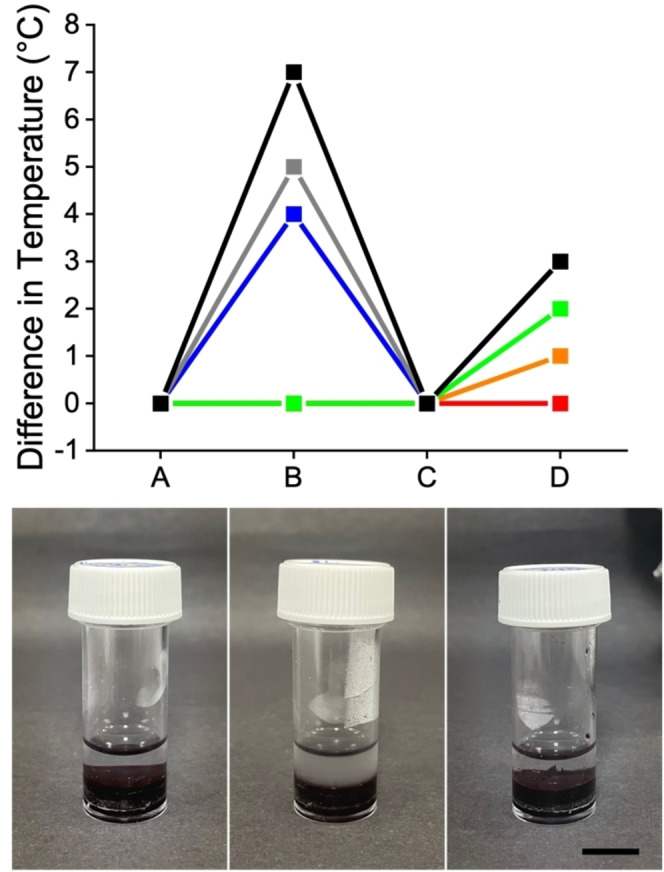
(Top) Temperature difference when irradiating PBI‐A gels with a 365 nm LED for 0 min (red); 30 seconds (orange); 1 minute (green); 5 min (blue); 30 min (grey) and 1 h (black). Temperatures were measured at four time points: (A) before any irradiation; (B) immediately after irradiation with the 365 nm LED; (C) after ten minutes of cooling post‐irradiation with the 365 nm LED; (D) after 15 min of irradiation with a 810 nm LED. (Bottom) Left to right shows a PBI‐A gel pre‐irradiated with a 365 nm LED and cooled with a PNIPAAm solution placed on top, the same gel after irradiation for 3 min with a near‐IR LED leading to heating of the PNIPAAm solution above the LCST, and a control sample without the pre‐irradiation with 365 nm LED but still with 3 min irradiation with the near‐IR LED showing no LCST transition. The scale bar represents 1 cm.

The increase in temperature when irradiating with the 810 nm near‐infrared light is controlled by the concentration of radical anion present, which in turn is controlled by the length of time for which the gel is irradiated with UV light (Figure 3top and Figure S4 and Figure S5). Without any 365 nm irradiation, there is no increase in temperature when further irradiating with 810 nm. However, even a small amount of time irradiating with the 365 nm LED (30 seconds) is sufficient for the PBI‐A gel to heat up by 1 °C using the 810 nm LED. After 5 min of irradiation with the 365 nm LED, the gel heats up further with increasing 365 nm exposure (Figure 3top, time point B). However, the subsequent heating produced by the 810 nm is limited to 3 °C. This suggests that after 5 min of 365 nm exposure, the concentration of radical no longer increases on this scale. The degree of heating is also sensitive to the wavelength of the LED used for the second irradiation despite the radical anion persisting in all cases (Figure S6 and S7).

The photothermal gels can be used to heat a solution above the gel (Figure 3bottom). A PBI‐A gel was irradiated and allowed to cool to room temperature within a vial. Following this, water was placed on top of the gel. Subsequent irradiation with a near‐IR LED leads to the gel warming and the water above increasing in temperature. This can be used to induce transitions in the aqueous solution. We added a solution of poly‐(*N*‐isopropylacrylamide) (PNIPAAm) above a PBI‐A gel post irradiation with a UV light and cooling. PNIPAAm shows a lower critical solution temperature (LCST) which can be tuned by the addition of salts.^[16]^ We added sodium sulfate to tune the LCST to 24 °C.^[16]^ Heating the gel with a near‐IR LED results in warming of the PNIPAAm solution and induces the LCST which can be seen visibly by a change in turbidity (Figure 3bottom). As expected, no heating was observed when the analogous experiment was carried out with a PBI‐G gel (Figure S8).

**Figure 4:**
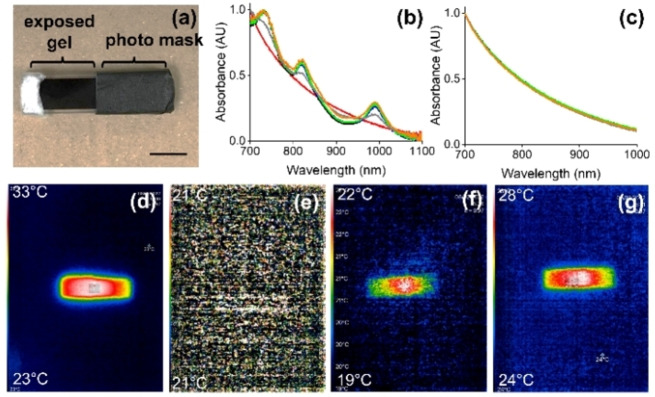
LED patterning of PBI‐A gels. (a) Photograph of gel formed in a 5 mm cuvette with light absorbing tape used as a mask; (b) normalised UV‐Vis data for the unmasked section of gel; (c) normalised UV‐Vis data for the masked section of the gel. For (b) and (c), data were collected before any irradiation (red); after 30 min of 365 nm irradiation (black); after 15 min relaxation (blue); after 30 min relaxation (green); after 1 h relaxation (grey); after 2 h relaxation (orange); (d) Photographs of the patterned gel using a thermal camera showing (i) the gel which has been irradiated with a 365 nm LED for 30 min; (ii) gel after cooling to room temperature. The mask was then removed after cooling; (iii) irradiation of the gel with an 810 nm LED for 1 min; (iv) irradiation of the gel with an 810 nm LED for 10 min. Scale bars for (d)–(g) represent 1 cm.

The gel can be patterned by exposing only part of the gel to UV light (Figure 4a). This results in localized formation of the radical anion (Figure 4b and 4c). Once the radical anion has formed, it is stable for an extended period of time during which there is no evidence for loss of patterning. However, when the sample is irradiated after 2 h with an 810 nm LED, the whole gel warms up (Figure 4d). This is perhaps as expected as the water is mobile within the gel network even if the PBI network, and so the radical anion, itself is immobile. This may also be down to the geometry used in this experiment (see below). We emphasize that the radical anions here are stable even in the presence of oxygen; the aggregated structure provides stability meaning that the effects described here can occur even in non‐degassed water and with no need to preclude the presence of oxygen.

**Figure 5:**
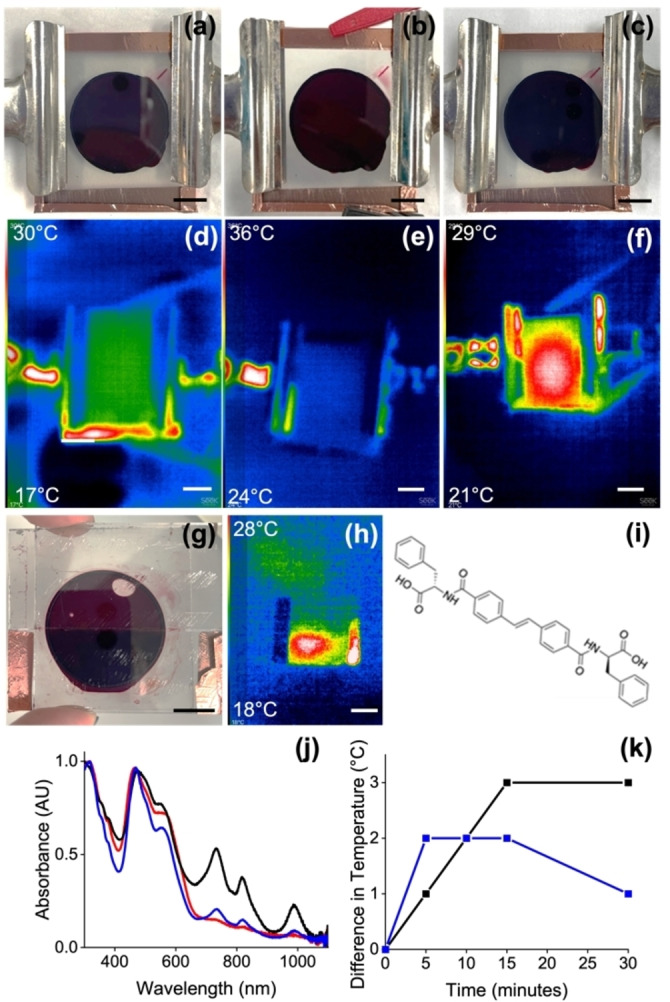
(a) Photograph of PBI‐A gel on an electrode; (b) gel after formation of the radical anion; (c) gel after electrochemical formation of the radical anion followed by irradiation with 810 nm LED. (d)–(f) show the same gels imaged using a thermal camera; the upper temperature achieved is at the top left and the minimum temperature on each image in the bottom left; (g) photograph of an electrochemically‐patterned gel and (h) the corresponding thermal image after irradiation with a 810 nm LED; (i) Chemical structure of Stilbene‐F; (j) normalised UV‐Vis spectrum of a mixed PBI‐A and Stilbene‐F gel before any irradiation (red), after 10 min of 365 nm radiation (black) and after 10 min of 450 nm radiation (blue); (k) temperature difference when irradiating mixed PBI‐A and Stilbene‐F gels with 810 nm. Black data shows gel which was first irradiated with 365 nm and cooled and blue data shows gel which was first irradiated with 450 nm and cooled before the 810 nm irradiation. Scale bars for (a) represent 1 cm.

However, a potential issue here with this approach is the need to irradiate first with UV light to generate the radical anion. First, this leads to simultaneous heating which may limit applications if one wishes to use the photothermal effect to trigger a process using heat. Second, UV light can cause damage to some systems. Here, we show that there are two methods to successfully overcome this issue. First, the radical anion can also be formed electrochemically. PBI‐A gels were prepared on an electrode surface in a mold. After this, the radical anion was formed by electrochemical reduction, resulting again in the peaks in the UV‐Vis spectrum for the radical anion. These gels do not heat up during formation of the radical anion, showing that the initial heating in the cases above is entirely due to the high energy UV light. The radical anion is also not air‐sensitive when formed electrochemically and persists for at least hours. Irradiation with an 810 nm LED after the electrochemical formation of the radical anion results in the gel warming up (Figure 5a–f). A patterned electrode can be used to locally form the radical anion. In this case, irradiation with an 810 nm LED results in more localised heating (Figure 5g) than in the case described above where the radical anion was formed using UV light. The reason for this is not clear but may be down to heat transfer along the quartz in the case of the cuvette as opposed to the thicker gel used for the electrochemical approach.

As a second method to avoid using UV light to generate the radical anion, a multicomponent system can be used. Gelling PBI‐A in the presence of a stilbene‐based gelator, Stilbene‐F (Figure 5i) results in a self‐sorted system being formed.^[17]^ We have previously shown that this self‐sorted system results in the self‐assembled fibers formed from Stilbene‐F being able to donate an electron to fibers formed from PBI‐A; this means that the radical anion can now be generated by irradiation with 450 nm light instead of UV light.^[17]^ We believe that this is due to self‐assembled fibers formed by PBI‐A and fibers formed by stilbene‐F in the multicomponent gel and in contact with one another, allowing donation of an electron from the stilbene‐F to PBI‐A. This means a lower energy wavelength is required to generate the radical anion since the electron is donated from the stilbene. Gels were therefore prepared from this multicomponent system, and irradiation with a 450 nm LED results in the formation of the characteristic peaks for the radical anion (Figure 5j). Again, further irradiation with the 810 nm NIR LED results in warming due to the photothermal effect (Figure 5k; further information in Figures S9–S11). Using the current set‐ups, the absolute heating is lower than when we use 365 nm light to form the radical anion. However, we are not directly comparing gels of the same size and thickness due to experimental protocols. From this perspective, we would not directly compare absolute efficiency here, but rather the observation that the higher wavelength light can be used to generate the radical anion and hence allow the system to exhibit the photothermal effect.

Finally, we note again the need for tuning of the system. A number of other PBIs that form the radical anion in the aggregated gel state, including PBI‐L, PBI‐F, PBI‐V and PBI‐H. The formation of the radical anion in each of these systems results in the appearance of new bands in the UV‐Vis spectra in the region of 700 nm or greater (Figure S12, Supporting Information). Interestingly, of these, only PBI‐F and PBI‐L show the photothermal effect (Figure S13), showing that success in such systems is not a straightforward as might be expected. We hypothesize that this is down to the packing modes within the self‐assembled aggregates. The molecular packing is generally probed by UV‐Vis spectroscopy. The spectra for the PBIs here show differences in terms of the peak ratios, the 0‐0/0‐1 vibronic band intensity ratio which is influenced by the aggregation of PBI molecules.^[18]^ However, there is no clear link between the ratios and whether or not the PBI shows the photothermal effect in the gel phase. This may therefore reflect differences in the aggregation of the supramolecular fibers or the network formed, perhaps allowing a means of losing energy by some other means on irradiation with the near‐IR LED. This is the focus of further work.

## Conclusions

We have shown that we can prepare gels from a perylene bisimide which exhibit the photothermal effect when the radical anion is present. The radical anion can be formed using UV light, 450 nm light (for a multicomponent system) or electrochemically. For specific perylene bisimides, the formation of the radical anion results in new absorption peaks in the near‐IR, meaning that subsequent irradiation with a near‐IR LED results in the gel warming. This can be used for example to heat a solution above the gel. The degree of warming can be controlled by the concentration of radical anion present. We emphasize again that these systems are stable in the presence of oxygen, meaning that there is significantly less restriction in terms of their use as compared to many other molecular based PBI systems where oxygen quenches the radical anion and hence the photothermal effect.

## Experimental Section

**Synthesis**: PBI‐A, PBI‐L, PBI‐F, PBI‐H and PBI‐V were synthesized using previously reported methods.[^10^, ^13^] The amino acid functionalized Stilbene‐F was synthesized using previously reported methods.^[19]^ The synthesis of PBI‐G is described below. Analytical data and spectra for all PBIs used here are in the Supporting Information.

PTCDA (3.88 g, 9.9 mmol) was reacted with L‐glycine (1.49 g, 19.9 mmol) using molten imidazole (20.22 g, 297 mmol) as the solvent. The reagents were mixed thoroughly in a 100 mL Schlenk flask at room temperature and purged with nitrogen for 15 min. The mixture was then heated to 130 °C for 5 h while stirred with a magnetic stirrer bar under nitrogen. The temperature was then lowered to 90 °C, and deionized water (50 mL) was added. The solution was left to stir for 1 h and then cooled to room temperature. Once cooled, the solution was filtered through filter paper under gravity to remove any unreacted PTCDA. The filtrate was collected, and the pH was lowered to below pH 4 with 2 M HCl (50 mL), triggering precipitation of the PBI. The precipitate was collected by vacuum filtration, washed with 2 M HCl (60 mL) and then thoroughly with deionized water (60 mL) until a neutral pH was achieved. The resulting solid was dried under vacuum. The solid was then washed in refluxing 2 M HCl (50 mL) for 5 h; the solid was collected by vacuum filtration and washed three times with 2 M HCl (10 mL) and three times with deionized water (10 mL). The dark red solid was dried under vacuum to give a blood‐red solid as the product. A typical yield obtained for all PBIs was between 80–90 %.

**Preparation of PBI solutions**: PBI solutions were prepared at a concentration of 5 mg/mL at room temperature. The required PBI was weighed out into a 50 mL Falcon tube alongside one molar equimolar of 0.1 M NaOH solution with respect to PBI, and the final volume made up with water to give the desired 5 mg/mL PBI concentration. The solutions were then stirred overnight at 1000 rpm on a stirrer plate. The next day, the pH of the solutions was adjusted to 9.0±0.1.

**Preparation of mixed PBI‐A and Stilbene‐F solutions**: PBI solutions were prepared at a concentration of 10 mg/mL at room temperature. PBI was weighed out into a 50 mL Falcon tube alongside one molar equimolar of 0.1 M NaOH solution with respect to PBI, and the final volume made up with water to give the desired 10 mg/mL PBI concentration. The solutions were then stirred overnight at 1000 rpm on a stirrer plate. The next day, the solutions were adjusted such that the pH was 9.0±0.1. Stilbene‐F solutions were prepared at a concentration of 10 mg/mL at room temperature. Stilbene‐F was weighed out into a 50 mL Falcon tube alongside two molar equivalents of 0.1 M NaOH solution with respect to Stilbene‐F, and the final volume made up with water to give the desired 10 mg/mL Stilbene‐F concentration. The solution was then stirred overnight at 1000 rpm on a stirrer plate. The next day, the solution was adjusted such that the pH was 9.0±0.1. Once at the correct pH, the two single‐component solutions were then stirred together at 1000 rpm overnight in equal volumes to provide a solution with a final concentration of 5 mg/mL of each gelator. The pH was again adjusted to pH 9 if needed after mixing the two systems.

**Preparation of PolyNIPAAM solutions**: Poly(N‐isopropylacrylamide) was purchased from Sigma‐Aldrich with a molecular weight of 20,000–40,000 Da. A solution was prepared in deionized water at a concentration of 10 mg/mL. To this, Na_2_SO_4_ was added such that the concentration was 10 mg/mL. The lower critical solution temperature (LCST) was checked using turbidity to be 23–24 °C. 1 mL of this solution was placed on top of a pre‐formed PBI‐A gel (1 mL) which had either been pre‐irradiated with a 365 nm LED as above or had not been pre‐irradiated. A 740 nm LED was placed underneath the gels and the gel irradiated for 3 min.

**Gelation**: Gelation of the PBI solutions was carried out using glucono‐δ‐lactone (GdL). 2 mL of solution was added to a 7 mL Sterilin vial with 20 mg GdL and shaken 5 times to give a final PBI concentration of 5 mg/mL and GdL concentration of 10 mg/mL. If preparing a gel for a cuvette, the required volume was pipetted into the cuvette at this time, the cuvette sealed, and then left overnight to gel. If preparing a gel for a vial, the vial was left undisturbed overnight after shaking 5 times. For mixed PBI‐A and Stilbene‐F gelation, the same procedure was used but now with 20 mg/mL GdL due to the increase of gelation concentration overall.

**Electrochemical Reduction**: Gels used for electrochemical reduction were prepared as described above but using a 0.1 M NaCl background electrolyte. Gels were formed in a 4 cm O‐ring with a diameter of 1 mm in between two pieces of FTO coated glass facing inwards held in place with bulldog clips. Copper tape was placed on opposite ends of the top and bottom pieces of glass as working and counter electrode. For the patterned gel, a line was scored down the center of the conductive side of the glass with a glass scribe, and copper tape was only applied to one half of the glass, rendering the other side not conductive. The gels were reduced in a two‐electrode configuration (no reference electrode) using a Palmsens potentiostat. A potential of −0.7 V was applied for 3 min to form the radical anion. Thermal and optical images were taken at this point. Then a near‐IR LED was used to irradiate the gel for 5 min. Again, themal and optical images were captured.

**LEDs**: LEDs of various wavelengths were purchased from RS Components Ltd and used inside a UV box or under a UV blanket. The distance from the LED to the sample was adjusted and measured using an intensity sensor (Thorlabs Optical Power Meter PM100D and Thorlabs sensor S/N : 16100711) such that the intensities used for all the LEDs was 16 mW.

**pH**: A pH probe (Hanna, FC200) was calibrated with pH 4, pH 7 and pH 10 buffers for measurements. The reported accuracy of the pH probe is±0.1.

**Ultraviolet‐Visible Absorption Spectroscopy**: UV‐Vis absorption data were obtained using an Agilent Technologies Cary 60 UV‐Vis spectrophotometer. Spectra were collected at a scan rate of 2 nm s^‐1^. Gels were prepared such that they gelled overnight in a Hellma 0.1 mm demountable quartz cuvettes, with the exception of patterning experiments which were performed in 5 mm Starna scientific standard rectangular quartz cuvettes. This was done on a larger scale to try and emphasize any changes in temperature during patterning. All PBI‐A gel data is normalized to the maximum peak found at approximately 470 nm using:

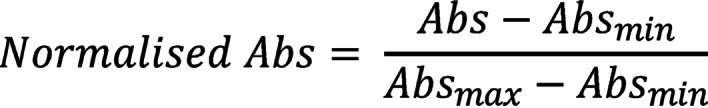




**Thermal Camera**: A Seek Thermal Compact Pro camera was used with the Seek Thermal mobile app (Version 2.2.8.0 for iOS) with either an iPhone 6s Plus or an iPhone 7. JPEGs were produced as outputs. The thermal sensor has a resolution of 320 by 240 pixels giving 76,800 temperature pixels. The detection distance is quoted as 6 inches (15 cm) to 1,800 feet (550 meters) with a 32° field of view. The temperature range is −40 °F (−40 °C) to 626 °F (330 °C) with a frame rate of <9 Hz and an adjustable focus. The thermal sensitivity is quoted as <70 mK with a spectral range of 7.5 to 14 microns. To minimize glare from surroundings (such as laboratory ceiling lights), samples were placed underneath an upturned box which was adapted with a hole to allow only the lens of the camera to view the samples in question. This helped to reduce glare which made samples appear hotter than they were. The box also had the added benefit of keeping the camera at a constant height of 25 cm.

ImageJ (version 1.53k) with plugin Color Profiler (version 1.2, https://imagej.nih.gov/ij/plugins/color‐profiler.html) provided the mean red‐green‐blue (RGB) values of a selected area. A 64 by 64‐pixel square was positioned at the centre of the cuvette in the JPEG image and RGB values obtained. This was then compared to RGB values on the thermal scale bar until a match was found, giving a corresponding temperature of the sample at the selected point.

## Conflict of interest

The authors declare no conflict of interest.

1

## Supporting information

As a service to our authors and readers, this journal provides supporting information supplied by the authors. Such materials are peer reviewed and may be re‐organized for online delivery, but are not copy‐edited or typeset. Technical support issues arising from supporting information (other than missing files) should be addressed to the authors.

Supporting Information

## Data Availability

The data that support the findings of this study are available in the supplementary material of this article.
