# Supplementary material for: Photothermal Perylene Bisimide Hydrogels
Source: Chemistry. 2023 May 10;29(37):e202300663. doi: 10.1002/chem.202300663 (PMC10946608; doi:10.1002/chem.202300663)
Supplement: Supplementary file 1 — Supporting Information [file CHEM-29-0-s001.pdf]

Chemistry–A European Journal

Supporting Information

Photothermal Perylene Bisimide Hydrogels

Lisa Thomson, Rebecca E. Ginesi, Daniel D. Osborne, Emily R. Draper,* and Dave J. Adams*

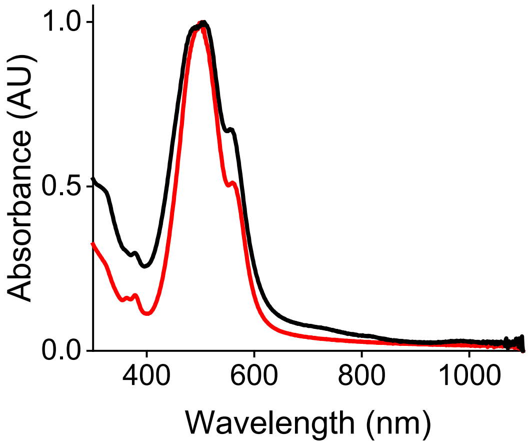

Figure S1. Normalised UV-Vis spectrum of a gel formed from PBI-G before (red) and after (black) irradiation with a 365 nm LED for 10 minutes.

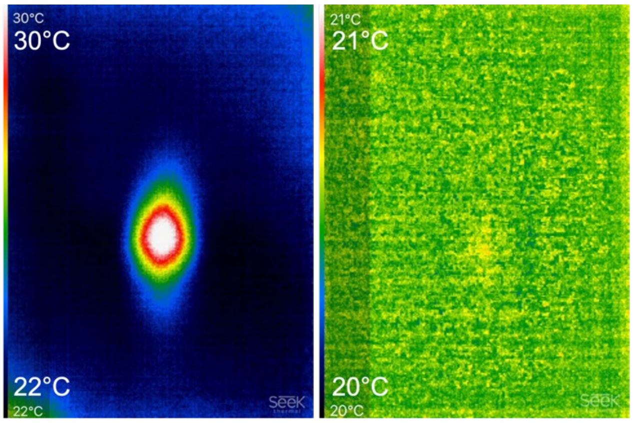

Figure S2. Photographs of gels formed from PBI-G using a thermal camera. In both cases, the upper temperature achieved is at the top left (red) and the minimum temperature on each image in the bottom left (dark blue). Left shows an image of a gel after irradiation for 10 minutes with a 365 nm LED. Right shows an image of the same gel after cooling for 10 minutes and then irradiated for 10 minutes with a 810 nm LED.

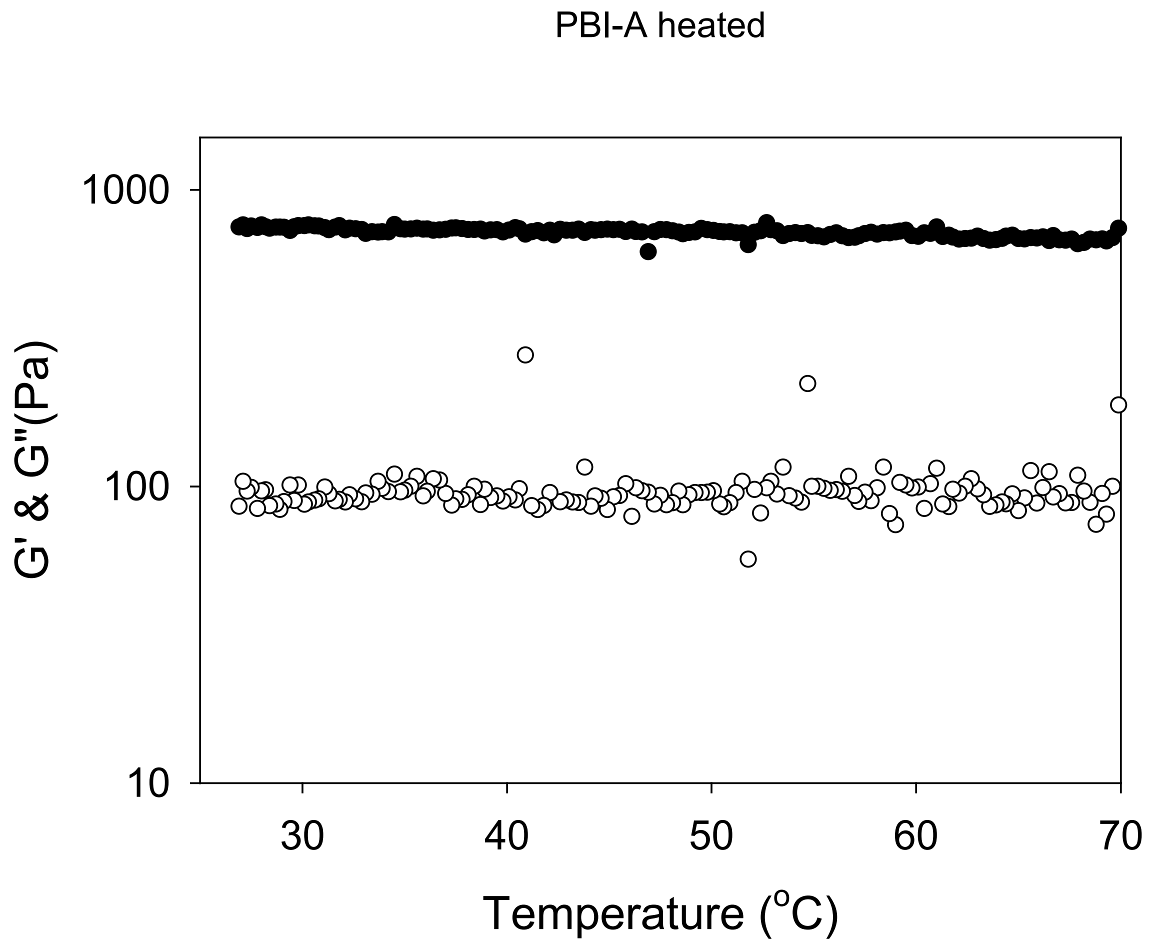

Figure S3. Temperature sweep data for a PBI-A gel showing that there is no melting until at least 70°C.

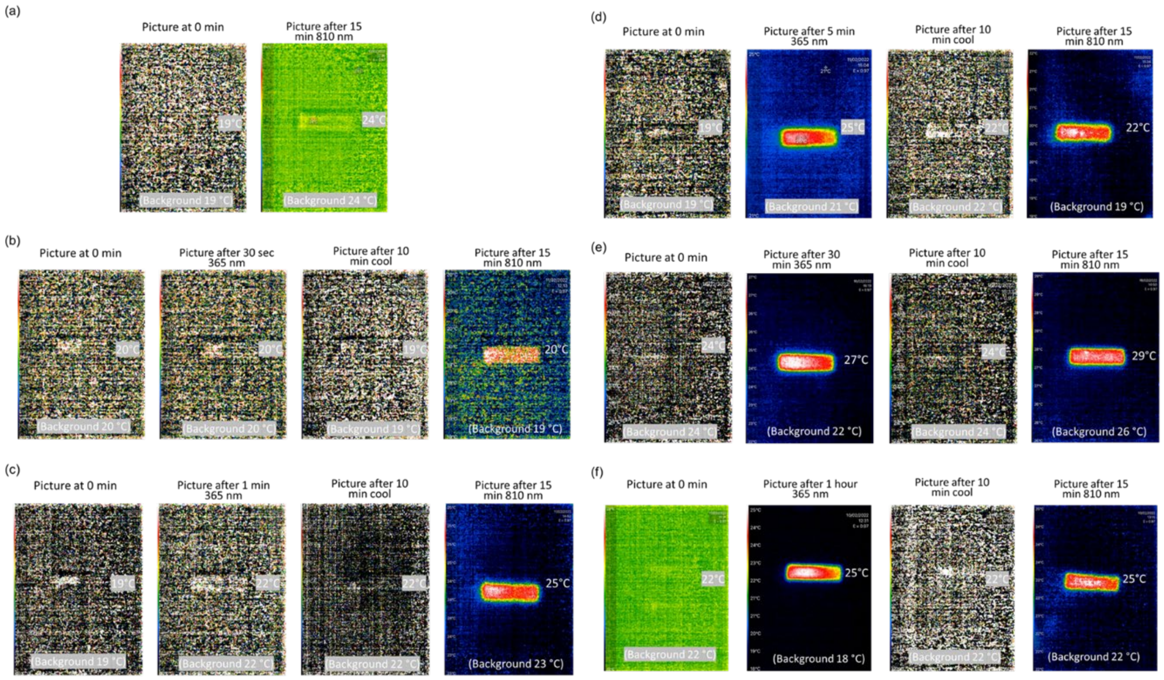

Figure S4. Processed thermal camera data showing different irradiation times of PBI-A gels in 0.1 mm cuvettes with the 365 nm LED at (a) 0 minutes; (b) 30 seconds; (c) 1 minute; (d) 5 minutes; (e) 30 minutes; (f) 1 hour of 365 nm irradiation. In all cases, samples were then cooled to room temperature, followed by 15 minutes of irradiation with an 810 nm LED.

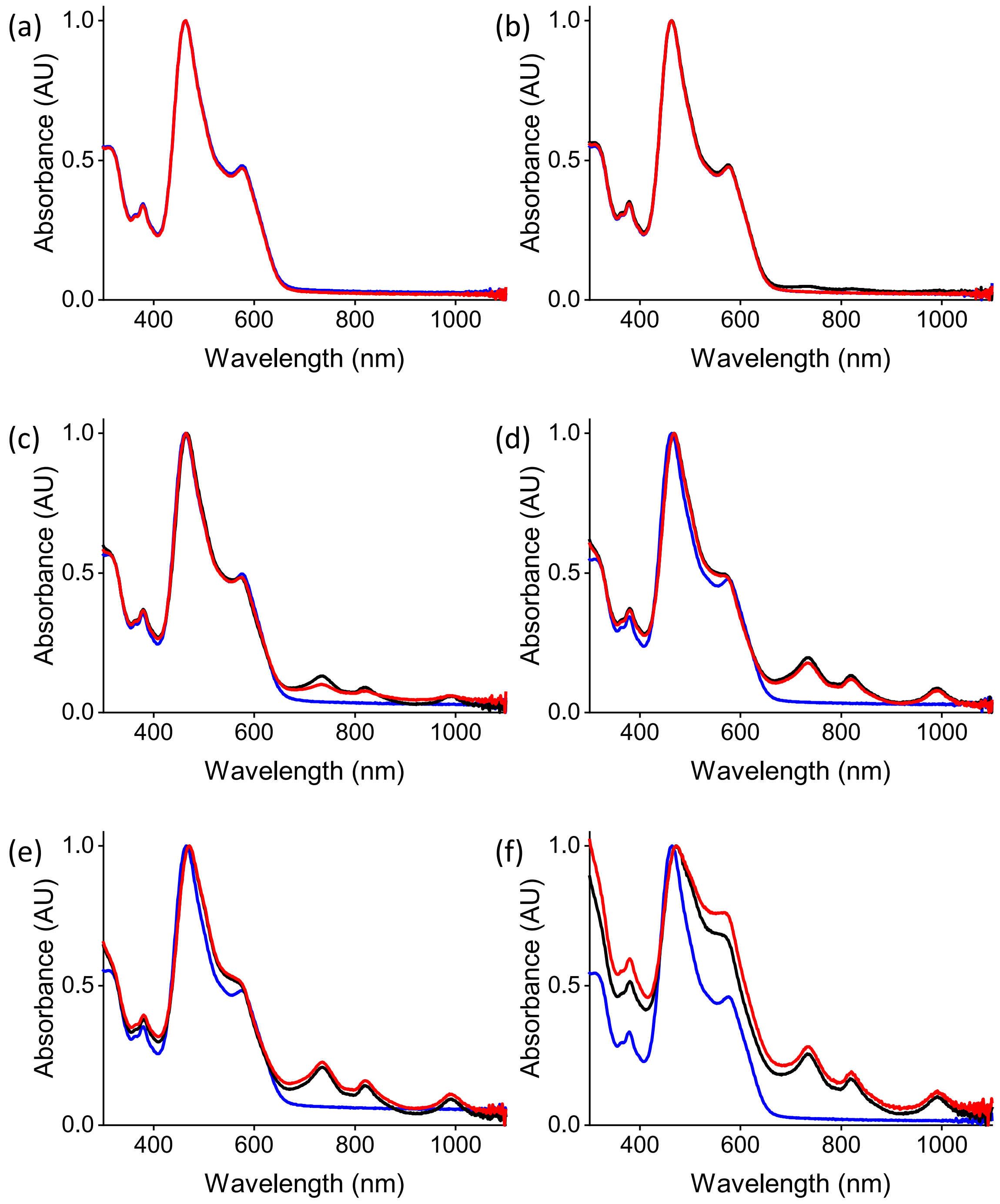

Figure S5. Normalised UV-Vis spectra of PBI-A gels in 0.1 mm cuvettes that were irradiated with a 365 nm LED (black) for (a) 0 minutes; (b) 30 seconds; (c) 1 minute; (d) 5 minutes; (e) 30 minutes; (f) 1 hour. In all cases, this was followed by 15 minutes of irradiation with an 810 nm LED (red). A control spectrum showing the sample before any irradiation is shown in blue in all cases.

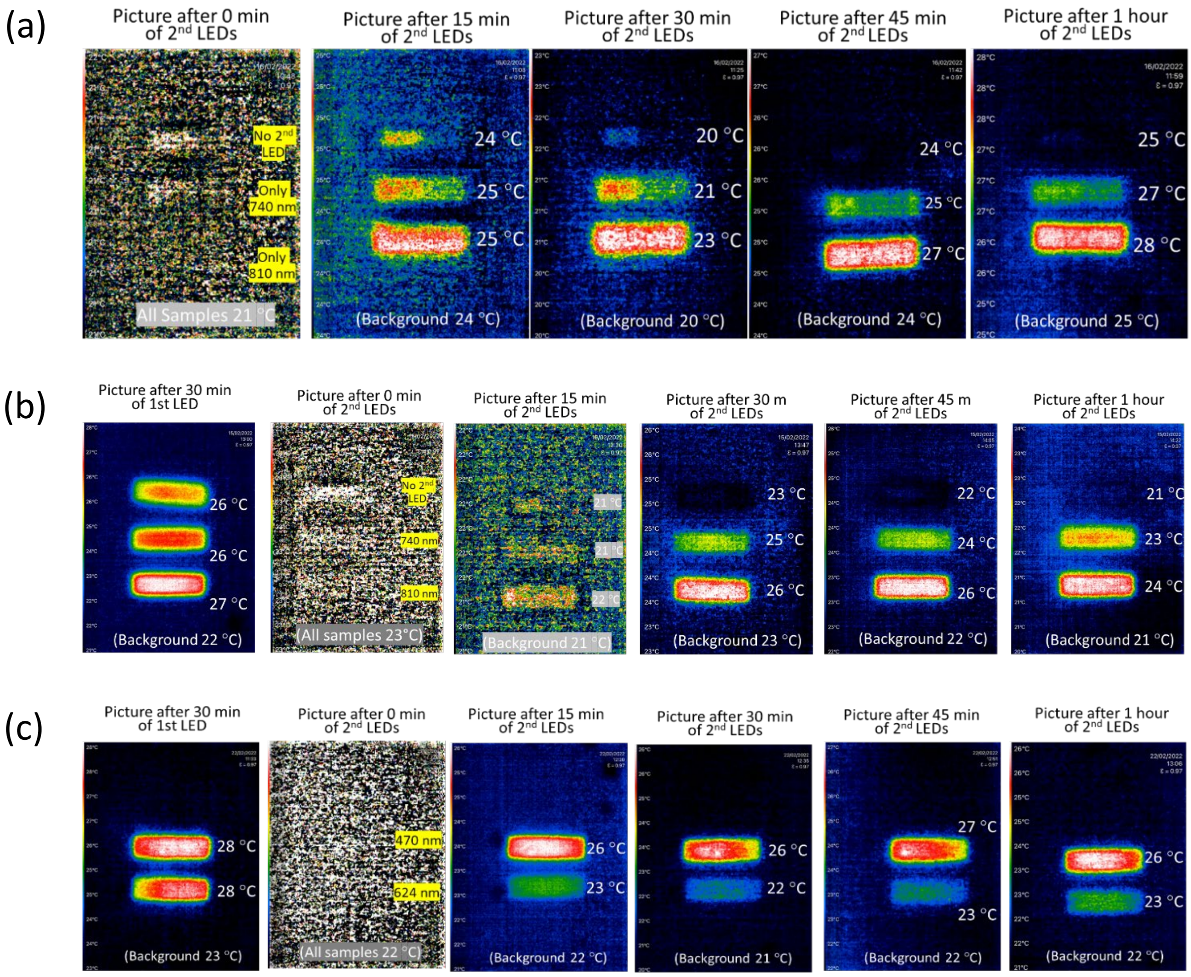

Figure S6. Processed thermal camera data of PBI-A in 0.1 mm cuvettes whilst varying the wavelength of LEDs used at various time intervals, showing (a) initially no 365 nm irradiation and therefore no radical present followed by (top cuvette) no 2nd LED, (middle cuvette) 740 nm LED, (bottom cuvette) 810 nm LED; (b) initially 365 nm irradiation for 30 minutes and therefore radical is present followed by (top cuvette) no 2nd LED, (middle cuvette) 740 nm LED, (bottom cuvette) 810 nm LED; (c) initially 365 nm irradiation for 30 minutes and therefore radical is present followed by (top cuvette) 470 nm LED, (bottom cuvette) 624 nm LED.

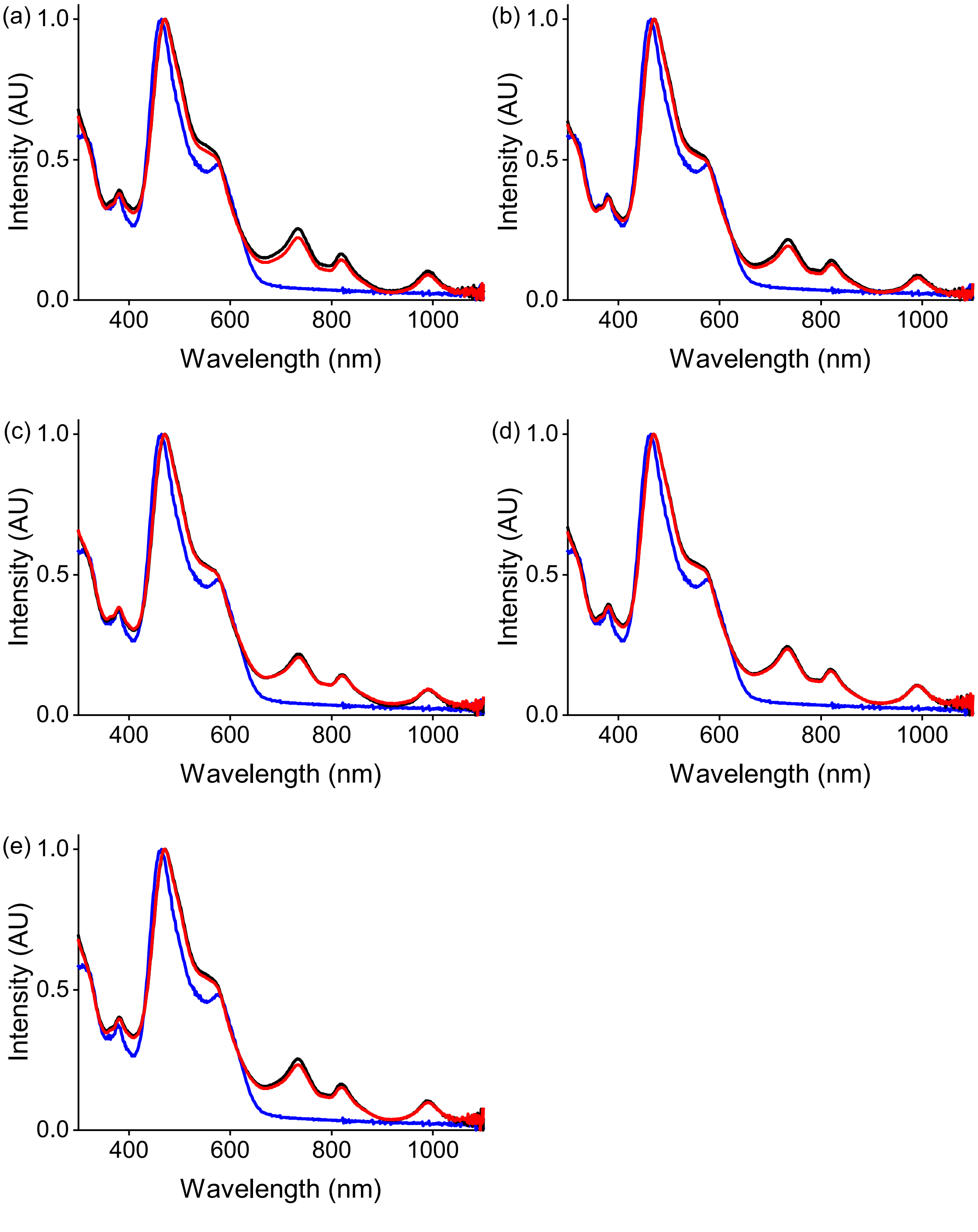

Figure S7. Normalised UV-Vis spectra of PBI-A gels in 0.1 mm cuvettes whilst varying the wavelength of the second LED. All samples were first irradiated for 30 minutes with 365 nm (black) and then irradiated for 1 hour (red) with (a) no additional radiation; (b) 470 nm; (c) 624 nm; (d) 740 nm; (e) 810 nm. A control spectrum showing the sample before any irradiation is shown in blue in all cases.

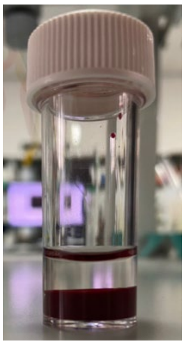

Figure S8. A PBI-G gel pre-irradiated with a 365 nm LED and cooled with a PNIPAAm solution placed on top and then irradiated for 3 minutes with a 810 nm LED showing no LCST transition.

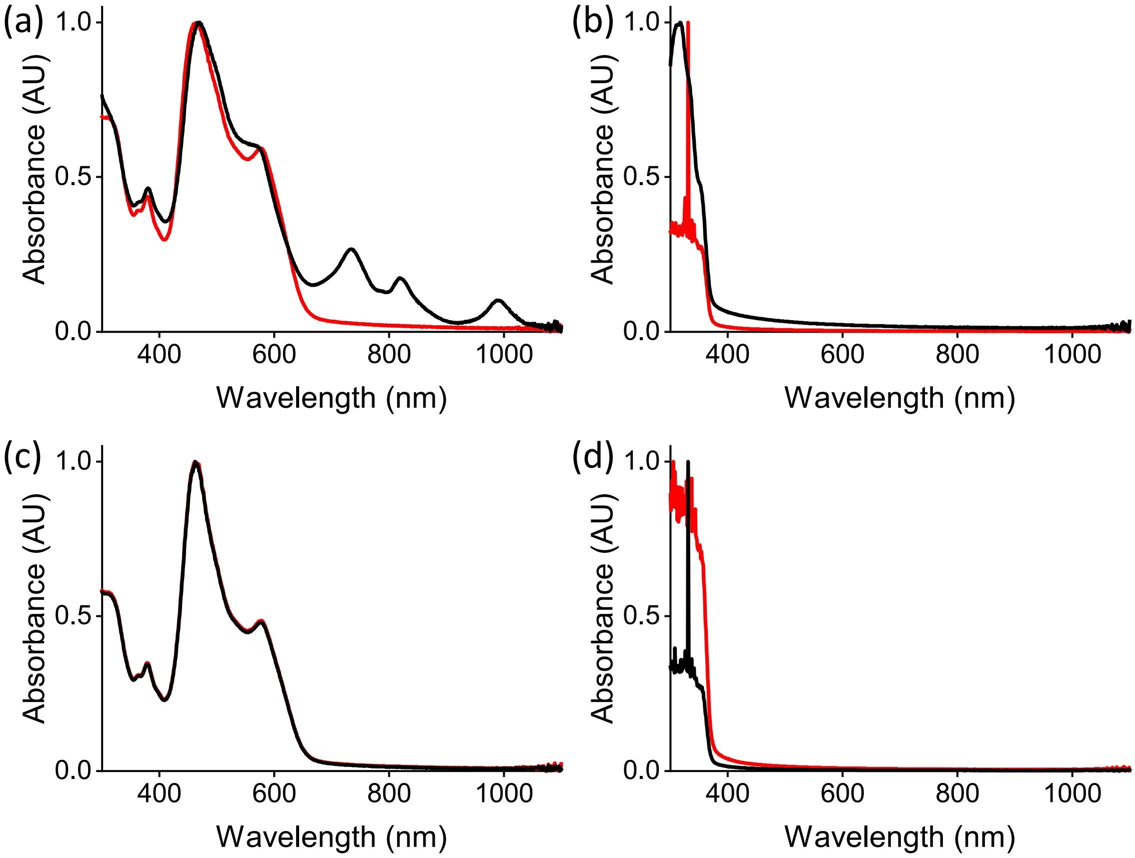

Figure S9. Normalised UV-Vis spectra for the single components of the mixed PBI-A and Stilbene-F gels showing in black (a) PBI-A gel at a concentration of 10 mg/mL irradiated with a 365 nm LED for 10 minutes; (b) Stilbene-F gel at a concentration of 10 mg/mL irradiated with a 365 nm LED for 10 minutes; (c) PBI-A gel at a concentration of 10 mg/mL irradiated with a 450 nm LED for 10 minutes; (d) Stilbene-F gel at a concentration of 10 mg/mL irradiated with a 450 nm LED for 10 minutes. A control spectrum of each component with no irradiation is shown in red.

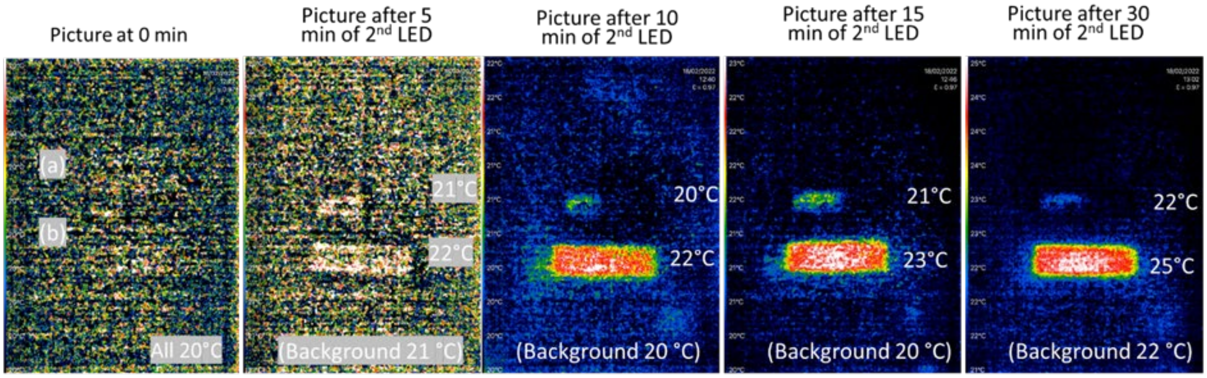

Figure S10. Mixed PBI-A and Stilbene-F gels whilst first irradiating with a 365 nm LED. Samples (a) and (b) were first irradiated for 30 minutes with the 365 nm LED and then allowed to cool. Sample (a) was not irradiated any further whilst (b) was irradiated with an 810 nm LED which increased the temperature of the gel.

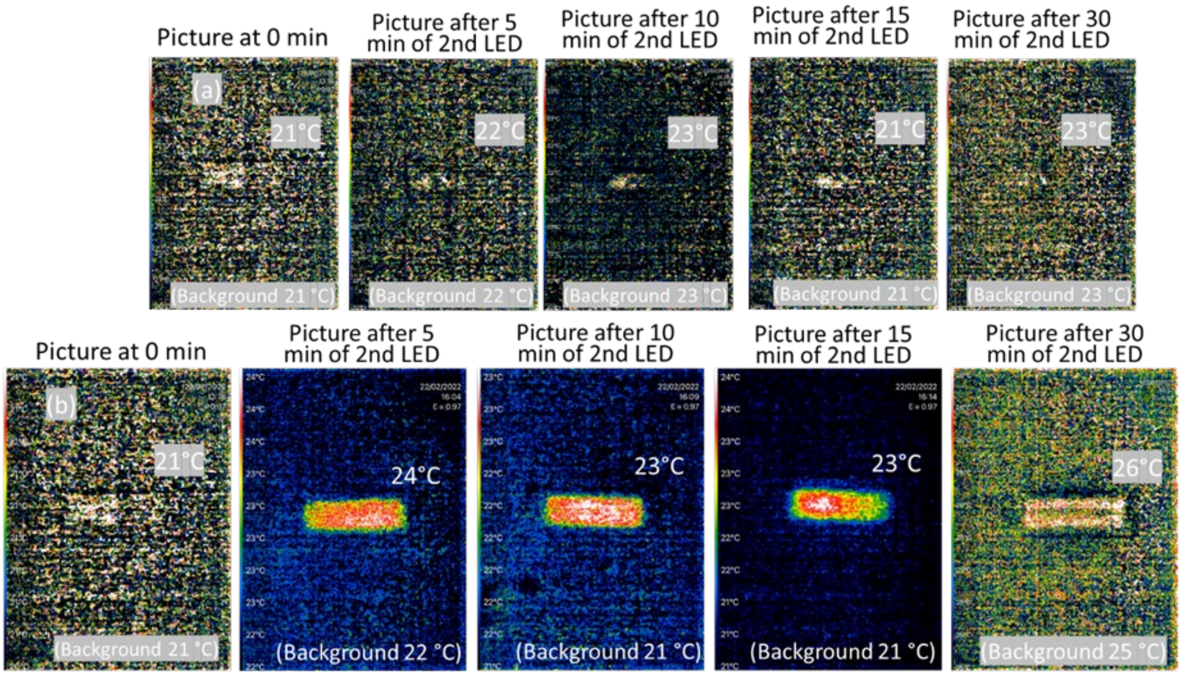

Figure S11. Mixed PBI-A and Stilbene-F gels whilst first irradiating with a 450 nm LED. Samples (a) and (b) were first irradiated for 30 minutes with the 450 nm LED and then allowed to cool. Sample (a) was not irradiated any further whilst (b) was irradiated with an 810 nm LED which increased the temperature of the gel.

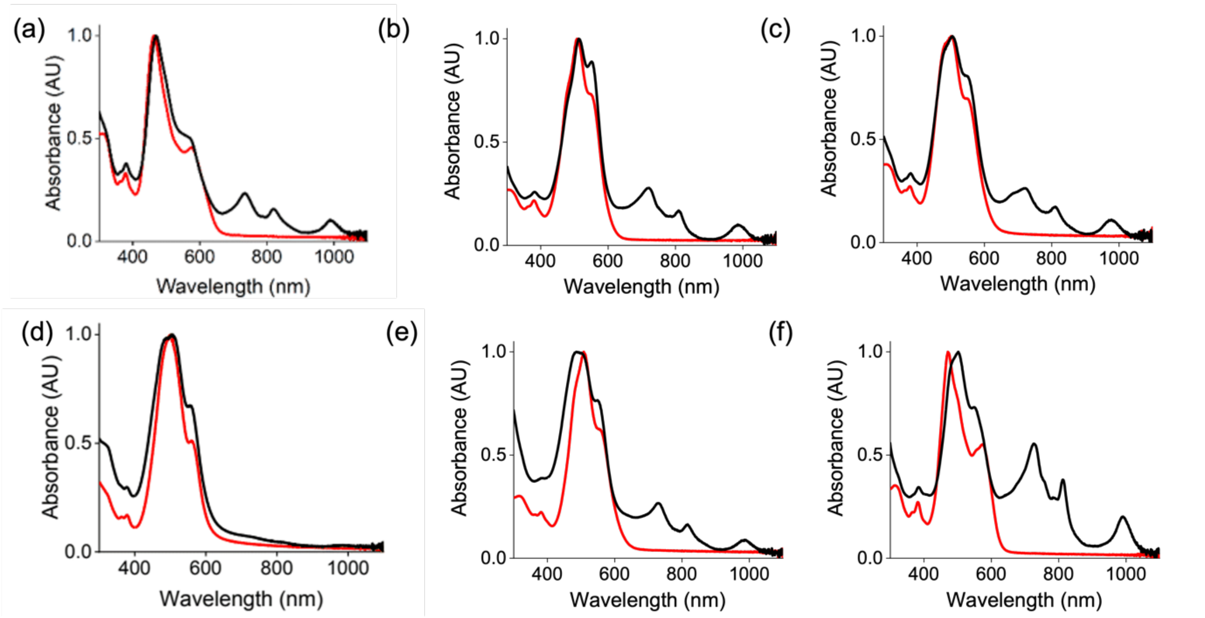

Figure S12. Normalised UV-Vis spectrum of a gel formed before (red) and after (black) irradiation with a 365 nm LED for 10 minutes for (a) **PBI-A**; (b) **PBI-F**; (c) **PBI-L**; (d) **PBI-G**; (e) **PBI-H**; (f) **PBI-V**.

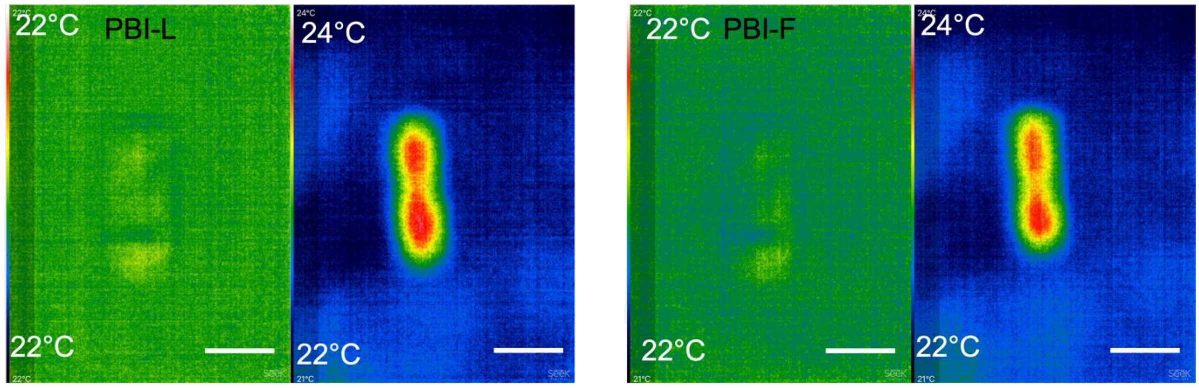

Figure S13. Photographs of gels formed from (left) PBI-L and (right) PBI-F using a thermal camera. In all cases, the upper temperature achieved is at the top left (red) and the minimum temperature on each image in the bottom left (dark blue). In both cases, the first image shows the gel cooled after irradiation with a 365 nm LED and right shows the image after 10 minutes of irradiation with a 810 nm LED. Scale bars represent 1 cm.

Analytical Data

PBI-G. ^{1}H NMR 400 MHz, (DMSO-*d*$_6$, 25°C): δ_H (400 MHz, DMSO-d$_6$, 80 °C) 8.91-8.74 (m, 4H, $\underline{H}_{Ar}$), 8.60-8.44 (m, 4H, $\underline{H}_{Ar}$), 4.79 (s, 4H, C$\underline{H}_2$). HRMS (ESI-) m/z: accurate mass calculated for $C_{28}H_{13}N_2O_8$: 505.06664. Found: 505.067774. FT-IR (cm^{-1}): 3004 (OH); 1696 (C=O); 1591 (C=O); 1331 (C-O); 1176 (C-O); 808 (aromatic C-H); 751 (aromatic C-H). Due to poor solubility, a ^{13}C NMR could not be obtained. PBI-G starts to decompose at around 420°C.

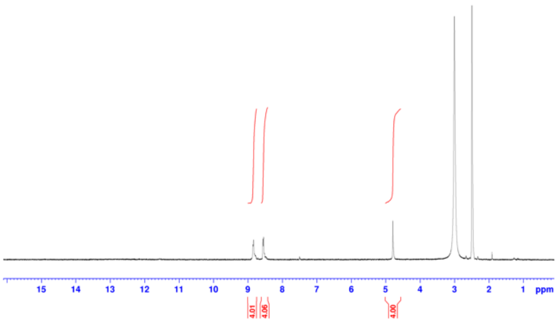

Figure S13. ^{1}H NMR spectrum for PBI-G in d$_6$-DMSO. The PBI-H is insufficiently soluble for a ^{13}C NMR spectrum to be recorded.

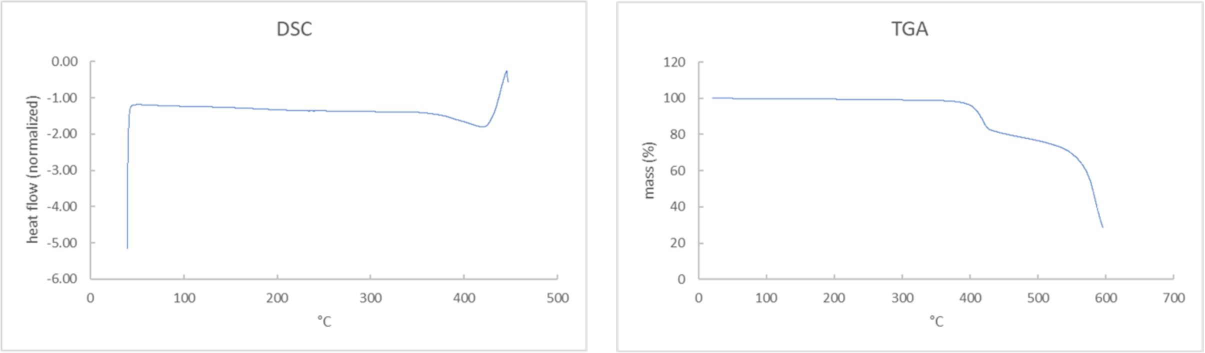

Figure S14. DSC (left) and TGA (right) of PBI-G.

PBI-A. ^{1}H NMR (400 MHz, DMSO-d_6, 25°C): 12.79 (2H, br s, $CO_2\underline{H}$), 8.27-8.16 (8H, m, $\underline{H}_{Ar}$), 5.61-5.56 (2H, m, $C\underline{H}^{*}$), 1.68 (6H, dd, *J* 6.94, 0.74, $C\underline{H}_3$). ^{13}C NMR (400 MHz, DMSO-d_6, 25°C): (125 MHz, DMSO-d$_6$, 80 °C) 170.54, and 161.54 ($\underline{C}$=O), 133.15, 130.27, 127.64, 124.61, 123.11, and 121.72 ($\underline{C}_{Ar}$), 48.43 ($\underline{C}H^{*}$), 14.12 ($\underline{C}H_3$).

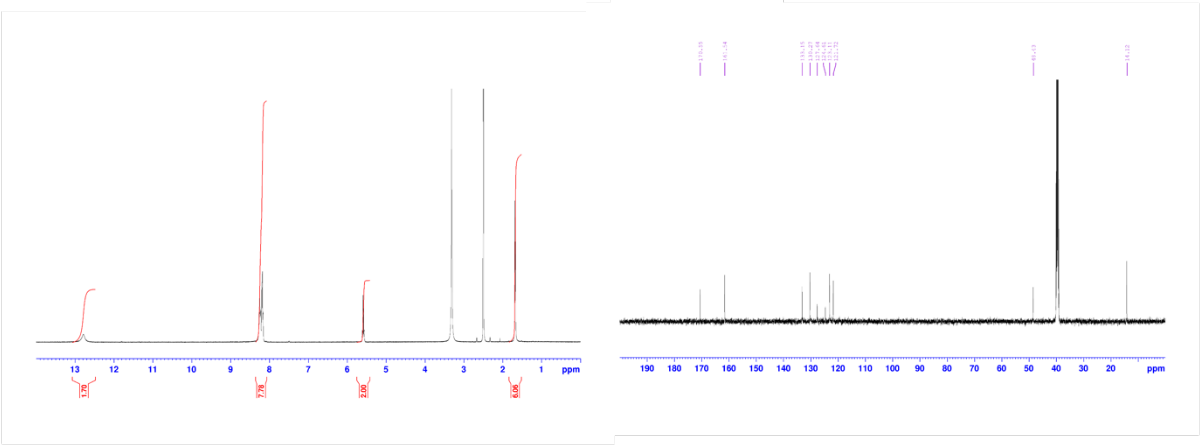

Figure S15. (Left) ^{1}H NMR and (right) ^{13}C NMR spectrum for PBI-A in d$_6$-DMSO.

PBI-L. ^{1}H NMR (400 MHz, DMSO-d_6, 25°C): δ (ppm) = 8.30 (8H, m, ArH), 5.58 (2H, t, *J* = 4 Hz, CH), 2.11 (4H, m, CH_2), 1.66 (2H, m, CH_2), 1.00 (6H, t, *J* = 4.8 Hz, CH_3), 0.97 (6H, t, *J* = 5.6 Hz, CH_3). ^{13}C

NMR (400 MHz, DMSO-d_6, 80°C): 170.5 (COOH), 161.9 and 161.9 (NC=O), 133.3, 133.2, 130.5, 130.4, 127.8, 127.8, 124.8, 124.68, 123.1, 123.0, 121.7, 121.7 (C_{Ar}), 51.4 (CH*), 37.7 (CH_2), 24.8 ($CH(CH_3)_2$), 22.3 and 21.7 ($CH(CH_3)_2$).

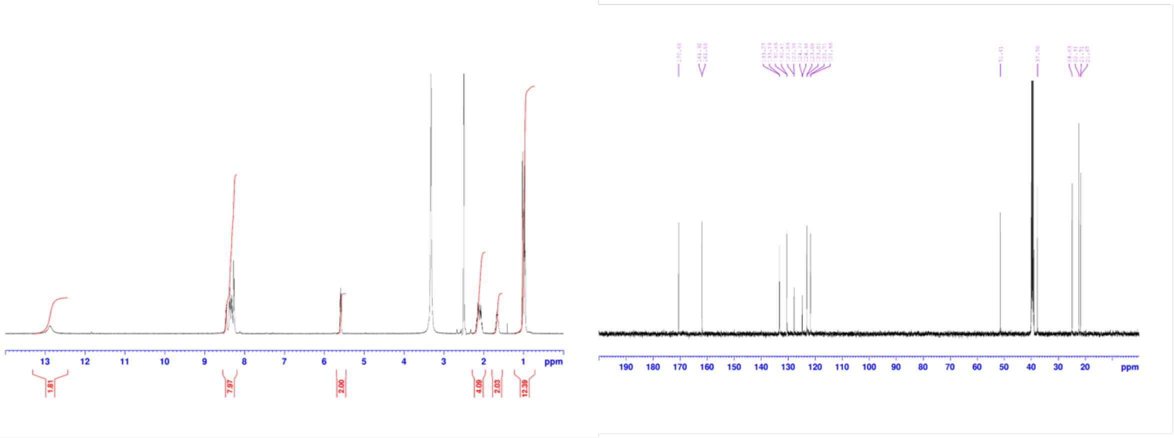

Figure S16. (Left) ^{1}H NMR and (right) ^{13}C NMR spectrum for PBI-L in d_6-DMSO. Most signals in the ^{13}C NMR spectrum are split due to restricted rotation effects. Acquiring the spectrum at 80 °C alleviates but does not eliminate this problem.

PBI-V. ^{1}H NMR (400 MHz, DMSO-d_6, 25°C): 12.83 (2H, br s, COOH), 8.54-8.50 (4H, m, H_{Ar}), 8.40-8.37 (4H, m, H_{Ar}), 5.20 (2H, d, *J* 9.16, CH*), 2.74 (2H, pseudo-dt, *J* 22.19, 6.81, $CH(CH_3)_2$), 1.28 (6H, d, *J* 6.44, $CH(CH_3)_2$), 0.80 (6H, d, *J* 6.88, $CH(CH_3)_2$). ^{13}C NMR (125 MHz, DMSO-d_6, 80 °C) 169.84 (C=O), 162.06 (C=O), 133.23 and 133.20 (C_{Ar}), 130.60 (C_{Ar}), 127.81 (C_{Ar}), 124.74 and 124.71 (C_{Ar}), 122.97 and 122.95 (C_{Ar}), 121.47 (C_{Ar}), 58.09 (C*), 26.89 ($CH(CH_3)_2$), 21.59 ($CHCH_3$), 18.74 ($CHCH_3$). Some carbon NMR signals are split due to restricted rotation around the C-N bonds or the presence of atropo-isomers. Acquiring the NMR at elevated temperature simplifies the spectrum by coalescing some of those signals.

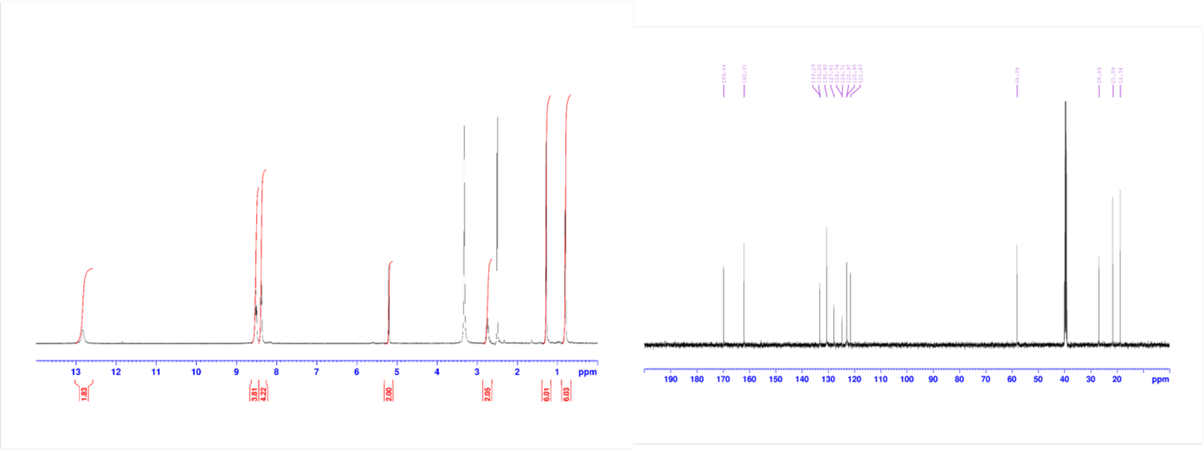

Figure S17. (Left) ^{1}H NMR and (right) ^{13}C NMR spectrum for PBI-V in d_6-DMSO.

PBI-F. ^{1}H NMR (400 MHz, DMSO-d_6, 25°C): δ = 13.10 (2H, br s, COOH), 8.20 (8H, br s, H_{Ar} perylene), 7.26 (4H, d, *J* 7.44, Ph-H), 7.18 (4H, t, *J* 7.42, Ph-H), 7.08 (2H, d, *J* 7.24, Ph-H), 5.96 (2H, dd, *J* 9.70, 5.54, CH*), 3.64 (2H, dd, *J* 14.10, 5.46, $PhCH_aH_b$), 3.44 (2H, dd, *J* 14.02, 9.98, $PhCH_aH_b$). ^{13}C NMR (400 MHz, DMSO-d_6, 80°C): (100 MHz, DMSO-d_6) 170.68 (C=O), 161.83 (C=O), 137.93, 132.69, 130.45, 129.14, 128.20, 127.47, 126.51, 124.22, 122.38, and 121.31 (C_{Ar}), 53.94 (CH*), 34.35 ($PhCH_2$).

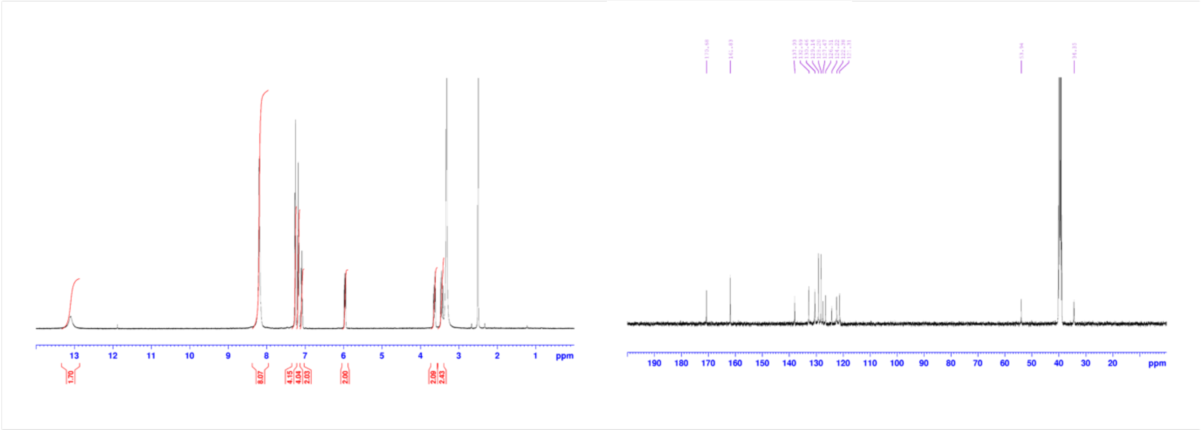

Figure S18. (Left) ^{1}H NMR and (right) ^{13}C NMR spectrum for PBI-F in d_6-DMSO.

PBI-H. ^{1}H NMR (400 MHz, DMSO-d_6 + TFA) 14.34 and 14.00 (broad, COO$\underline{H}$), 8.90 (2H, d, *J* 1.0, $\underline{H}_{Ar}$), 8.89-8.81 (4H, m, $\underline{H}_{Ar}$), 8.59-8.45 (4H, m, $\underline{H}_{Ar}$), 7.44 (2H, s, $\underline{H}_{Ar}$), 5.85 (2H, dd, *J* 9.6, 4.4, C$\underline{H}^{*}$), 3.70 (2H, dd, *J* 15.4, 4.1, C$\underline{H}_2$), 3.45 (2H, dd, *J* 15.5, 9.7, C$\underline{H}_2$).

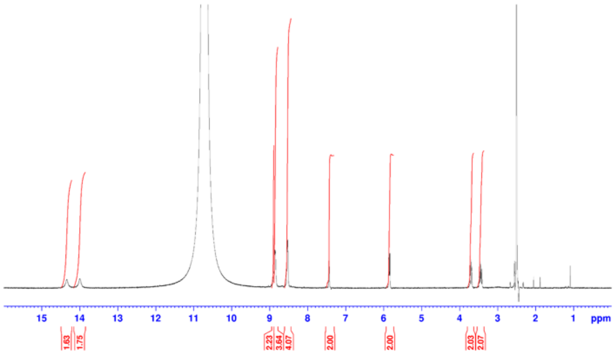

Figure S19. ^{1}H NMR spectrum for PBI-H in d_6-DMSO with added TFA to increase solubility. The PBI-H is insufficiently soluble for a ^{13}C NMR spectrum to be recorded.
